# Headaches attributed to airplane travel: a Danish survey

**DOI:** 10.1186/s10194-016-0628-7

**Published:** 2016-04-14

**Authors:** Sebastian Bao Dinh Bui, Torben Petersen, Jeppe Nørgaard Poulsen, Parisa Gazerani

**Affiliations:** SMI®, Department of Health science and Technology, Faculty of Medicine, Aalborg University, Aalborg, Denmark

**Keywords:** Headache, Airplane headache, Flight, Triptans, High-altitude headache, Danish

## Abstract

**Background:**

Airplane headache (AH) is a headache that occurs during take-off and landing. The pain is described as severe, unilateral, and located in the fronto-orbital region. This study aimed at investigating the incidence of AH among Scandinavian air-travelers, and to elucidating potential risk factors.

**Methods:**

An online, Danish-survey was developed. The questionnaire consisted of 14 questions and was distributed through the Facebook-pages of Scandinavian-airlines and interest organizations. Participants reached the questionnaire through a web-link.

**Results:**

Out of 254 responses, 89 noted that they suffered from headaches associated to airplane travel. Of the 89, 21 cases the headache was severe and limited to 30 min duration, as described in the ICH’s criteria of AH. The remaining 68 cases indicated that the headache lasted longer than 30 min. Our data demonstrated that High-Altitude Headache (HAH) is a risk factor for developing AH (*p* < 0.05). Triptans (19 %) and paracetamol (5 %) were reported effective to relieve AH.

**Conclusion:**

This study indicates that up to 8.3 % of the studied population suffered from AH, with a higher incidence in those with a history of HAH. Self-medication by triptans and paracetamol were reported effective for relieving AH.

## Background

Airplane headache (AH) is an intense, short lasting headache - usually lasts around 30 min [1], and is exclusively related to airplane travels. AH has a sudden onset and may occur at any time during flight; although, with a high incidence during take-off and landing, with the highest frequency during the landing phase [1]. AH is thought to affect a small part of the population [2]. However, with occupancy of around 70 % and more than 3.3 billion commercially available airplane seats, the Bnai Zion Medical Center estimates more than 100 million cases of AH annually [3]. Population-based investigations on AH, and its underlying mechanisms, treatments, prevention strategies, and risk factors are limited. Hence, further investigation to provide a clear picture that will eventually lead to better diagnosis and treatment is warranted.

The pathogenesis of AH has not been clarified yet and considering a diverse group of patients, with a broad range of symptoms, it is complicated to define a clear underlying mechanism(s). It has been suggested that barotrauma to the sinuses may lead to vasodilation of the cerebral arteries, which in turn may play an essential role in development of AH [2, 4–7]. Hence, it is reasonable to consider that airplane headache may also be linked to dysregulation occurring in the cerebral arteries [8]. Previous studies have shown a high psychological impact, caused by fear of future attacks, and stress of flying [6]. No report is present that focus on a North-European population [2, 4–7, 9–11]. Hence, the present study was designed to provide an overview of AH in a Northern-European population. The outcome of this study might provide new evidence on the incidence and risk factors of AH.

## Methods

### Study design and population

An online survey-based trial was designed to identify the incidence of AH among Scandinavian-air travelers, since no information was available regarding incidence or risk factors of AH in this population. The inclusion criteria admitted individuals with an age of ≥ 18 years, with a history of at least 3 flights (without restrictions on length of flight).

Participants were asked to rate their pain intensity on a scale of 1–10, where 1–3 was considered as mild headache, 4–7 reflected moderate headache, and 8–10 was considered as severe headache. Furthermore, the participants were asked to note their choice of medication as well as time of consumption.

### Survey and recruitment

Participants were recruited through advertisements posted on the Facebook-pages of Scandinavian-airline companies, and other interest organizations, with a link directing them to a custom-designed Danish-questionnaire. The questionnaire was available in the period from the 15^th^ October 2014 to the 1^st^ of December 2014 (a total of 48 days). The questionnaire was composed of 14 questions with adequate space provided for a supplementary or descriptive text. The first 6 questions focused on the demographical information of the participants, including nationality, age, gender, migraine and HAH. Other questions focused on AH and more specifically on its symptoms and co-morbidities, while others focused on the flight composition. The questionnaire was designed and made available through Google Docs. All questions were designed in a way that they had to be answered before continuing to the next question, thus securing an answer to each question.

### Statistical analysis

In the text and tables, data are presented as number of responses and percentages, except age, which is presented as mean +/− SD (standard deviation).

Statistical analysis was performed using SPSS (version 22.0, IBM, USA), and the level of significance was set at 0.05. A *t*-test was performed to determine if there was an age-difference between the headache group and the non-headache group. Fisher's Exact Test or chi-square was used to determine if gender distribution, migraine and HAH are significant risk factors of AH.

## Results

A total number of 254 Scandinavian-air travelers participated in this survey. Among those, 89 (35 %) identified themselves to be a victim of headache attributed to airplane travel. However, based on the current version of the ICH’s diagnostic criteria (version 3, beta) [1] for airplane headache, only 21 of them (8.3 %) met diagnostic criteria (i.e., headache lasts less than 30 min).

Therefore, two groups were defined for analysis of the collected data: AH group (*n* = 21) and the non-AH group (*n* = 233).

There was no statistical difference in the gender distribution between the AH and non-AH group. Overall, there was a tendency for more females responding to the questionnaire (≈62 %). The gender distribution is outlined in Table 1.

**Table 1:** Gender distribution in AH-group and non-AH group

	AH-group	Non-AH-group	Total
Females	12 (5 %)	146 (57 %)	158 (62 %)
Males	9 (4 %)	87 (34 %)	96 (38 %)
Total	21 (9 %)	233 (91 %)	254 (100 %)

*AH* airplane headache. The numbers represent number of responses. Percentages are given in brackets

The average age for the AH group was 39 ± 14 years (range: 19–67, *p* > 0.05), there was no statistical age difference between the genders.

In total, 62 participants (24.4 %) indicated diagnosed with migraine. There was no statistical difference in the distribution of migraine between the two groups (7 in AH group (33 %) and 55 in non-AH group (24 %)), for gender distribution see Table 2). However, migraine appears to be more frequent in the AH group, although the population size does not allow a conclusive consideration. Fifty-five participants (22 %) indicated that they suffered from HAH. There was a statistical difference in the distribution of HAH between the two groups (*p* < 0.001, 13 in AH group (62 %) and 42 (18 %) in non-AH group, for gender distribution see Table 3).

**Table 2:** Gender distribution and occurrence of migraine in AH group and non-AH group

	Migraine in AH-group	Migraine in Non-AH group	Total
Females	6 (50 %)	48 (33 %)	54 (34 %)
Males	1 (11 %)	7 (8 %)	8 (8 %)
Total	7 (33 %)	55 (24 %)	62 (24 %)

*AH* airplane headache. The numbers represent number of responses. Percentages are given in brackets

**Table 3:** Gender distribution and occurrence of HAH in AH group and non-AH group

	HAH in AH group	HAH in Non-AH group	Total
Females	9 (75 %)	34 (23 %)	43 (27 %)
Males	4 (44 %)	8 (9 %)	12 (13 %)
Total	13 (62 %)	42 (18 %)	55 (22 %)

*AH* airplane headache, *HAH* high-altitude headache. The numbers represent number of responses. Percentages are given in brackets

The onset of headache was equally divided between take-off (first 30 min) and landing (last 30 min) with 8 participants in each category (38 %). Five participants (24 %) reported that the headache occurred sometime in between of take-off and landing (see Table 4).

**Table 4:** Onset of headache as reported by participants in the AH-group

	Take-off (first 30 min)	Landing (last 30 min)	During the flight	Total
Females	5 (63 %)	3 (37 %)	4 (80 %)	12 (57 %)
Males	3 (37 %)	5 (63 %)	1 (20 %)	9 (43 %)
Total	8 (38 %)	8 (38 %)	5 (24 %)	21 (100 %)

*AH* airplane headache. The numbers represent number of responses. Percentages are given in brackets

The majority of AH participants (91 %) described their headache as unilateral, fronto-orbital or fronto-parietal. The headache was described mainly as “pressing” (43 %), but also pulsating (29 %) and stabbing (29 %). The intensity of headache was described as severe (57 %) or moderate (43 %).

When asked to provide a possible cause for their headache, changes in cabin pressure during take-off and landing was reported as the most possible cause of their AH (95 %).

The AH-group was divided into two subgroups: A medicated-group (24 %) and a non-medicated-group (76 %) (see Table 5). Among participants in the medicated-group, 1 (5 %) took paracetamol, and 4 (19 %) used triptans (Table 5). Among those who used triptans, 2 females and 1 male were diagnosed with migraine.

**Table 5:** Medication consumed by participants in the AH-group and its distribution (AH: airplane headache)

	No medication	Medication	Total
Females	9 (43 %)	3 (14 %)	12 (57 %)
Males	7 (33 %)	2 (10 %)	9 (43 %)
Total	16 (76 %)	5 (24 %)	21 (100 %)

The numbers represent number of responses. Percentages are given in brackets

The participants were asked to note any chronic diseases, to rule out possible co-morbidities, as well as whether or not they had been suffering from recent bouts of influenza. One case of asthma and one case of epilepsy were noted in the AH group.

## Discussion

The present study showed that up to 8.3 % (*n* = 21) of our study population suffered from AH. There might have been a skewed gender distribution due to the possibility of a mainly young audience using Facebook. Despite this, close to 40 participants were aged 60+, the average age was 38.2 years ± 15.8 (range 18–76). Although, larger population-based studies would be required to give a more precise estimate of the prevalence of AH in Northern Europe (i.e., Denmark). However, our findings are in line with findings from Southern Europe [2, 4, 6, 12]. Based on the current ICH’s classification, AH is short-lived - usually around 30 min experienced when ascending or descending - with severe stabbing pain, usually on one side of the head, near the eye. Although, the clinical symptoms reported in our population is consistent with this classification; we also found 68 participants that claimed that they suffer from AH, however, we could not classify them based on the diagnostic criteria of AH, but noted that their headache occurred only while flying. Whether or not this group or some individuals in this group can still be identified as AH is not clear now, due to inadequate information.

However, the pain in AH-group (*n* = 21) was experienced as stabbing in only 29 % of the cases. The grading of severity was moderate and the onset of the attacks was equally divided between take-off and landing. Five participants experienced headache during the cruising, but the onset of the headaches could potentially have started after the take-off phase. Our findings in the AH group are not completely in accordance with the literature, where the headache is usually rated as severe, with a jabbing, stabbing or sometimes pulsating quality, and mostly occurs during the landing phase [2, 4, 6, 12].

To identify potential risk factors of AH, we asked if the participants had experienced a cold or any other nasal congesting syndrome (that can be a cofounding factor). Two AH-participants noted cases of cold, however they indicated having AH is independent of cold.

The participants were also asked to note if they suffered from any chronic diseases to identify co-morbidities. Two chronic diseases, asthma and epilepsy, were listed by the participants in the AH group. Based on our data gender does not seems to be a risk factor for AH, although our data indicate a higher female AH-ratio, which is in contrast to previous studies suggesting a male dominance [4, 6, 13]. A skewed gender distribution is known from previous studies with a higher incidence in men than in women. A skewed gender distribution is also known from other types of headaches, such as tension type headache, where males have a higher risk than females [14]. However, the risk of migraines is three times higher in females than in males (18 % F, 7 % M) [15].

According to our findings AH primarily occurred during take-off, and landing. This is consistent with the previous findings [4, 6] and the ICH’s criteria for AH [1]. Changes in cabin pressure when an airplane is ascending or descending could affect those passengers with a highly sensitive baroreflex [16]. One study has demonstrated that migraine patients may have a highly sensitive baroreflex compared with healthy controls [17]. If a similar sensitivity exists in AH, this might pose a possible pathway for the development of AH. Sensitive baroreceptors and the link with migraine remains to be properly proven [17]. Another mechanisms might be through trigeminal nerve stimulation of the mucosal tissue. Cabin pressure may also affect cranial arteries, causing vasodilatation and headache. The vasodilatation might be caused by an imbalance between internal and external air pressure [4, 18, 19]. Cabin pressure may also affect hypersensitive cranial arteries, possibly cerebral arteries, thereby causing vasodilation and headache [10, 11]. Based on our results, HAH is a risk factor for AH. HAH is short lasting condition, that may occur during changes in altitude (primarily; ascending over 2500 meters), and often seen in activities such as mountain climbing [1]. HAH is felt on both sides of the head with an intensity ranging from mild to moderate [1] different from AH, which is unilateral and severe in intensity [6]. A previous study has shown that the intensity of HAH increases at higher altitude [20]. Several studies have measured changes in cabin pressure during a flight [21], and a linear coherence has been shown between altitude and cabin pressure [21]. Cabin pressure would normally decrease with around 8 hPa for each 300 meters, until the plane reaches an altitude of 2500 m, at which, the cabin pressure stabilizes at an average of 846 hPa (≈0.85 bar) for the rest of the flight [21]. The decreased pressure might lead to different degrees of hypoxia and changing pressure in body tissues and cavities [22, 23]. As such, these changes might contribute to the development of HAH and AH in the ascending phase. A study has recently shown a possible relation between AH and “mountain descending headache”, which is a condition with common symptoms comparable with AH [24]. We therefore speculate that both headaches are triggered by increasing atmospheric pressure during the descending phase, which will result in expanding the air in the cavities and thereby inducing the headache in the aforementioned phase. However, the mechanism(s) underlying HAH, mountain descending headache and AH remains to be identified [25].

Five participants of the AH group used medication (24 %) (prior to their flight) in an effort to prevent AH. One individual self-medicated with paracetamol and four took triptans. These medications were reported effective to prevent AH. Our results are in line with a former study that examined the effect of triptans by analyzing 5 flight travelers with AH who all used triptans during a flight, they all reported complete pain relief for the duration of the flight [5]. Triptans are reported to be the most effective when taken 30 min before a flight [5].

## Conclusion

This study found that up to 8.3 % of our study group suffered from AH with an increased risk in those with a history of HAH. Triptans and paracetamol were used to alleviate AH. Population-based studies are called for identification of global AH incidence. In addition, further prospective studies are highly recommended to clarify the mechanisms underlying AH, its triggers and prevention strategies.
