# Erratum to: Headaches attributed to airplane travel: a Danish survey

**DOI:** 10.1186/s10194-016-0643-8

**Published:** 2016-05-09

**Authors:** Sebastian Bao Dinh Bui, Torben Petersen, Jeppe Nørgaard Poulsen, Parisa Gazerani

**Affiliations:** SMI®, Department of Health science and Technology, Faculty of Medicine, Aalborg University, Aalborg, Denmark

After publication of the original article [1] it was brought to our attention that there remained a number of grammatical errors in the article, which the author was concerned should be corrected. This has now been updated on the Springer Open website, although it won’t be reflected on indexing sites such as PubMed. Please therefore refer to the Springer Open website for the corrections to this article.
